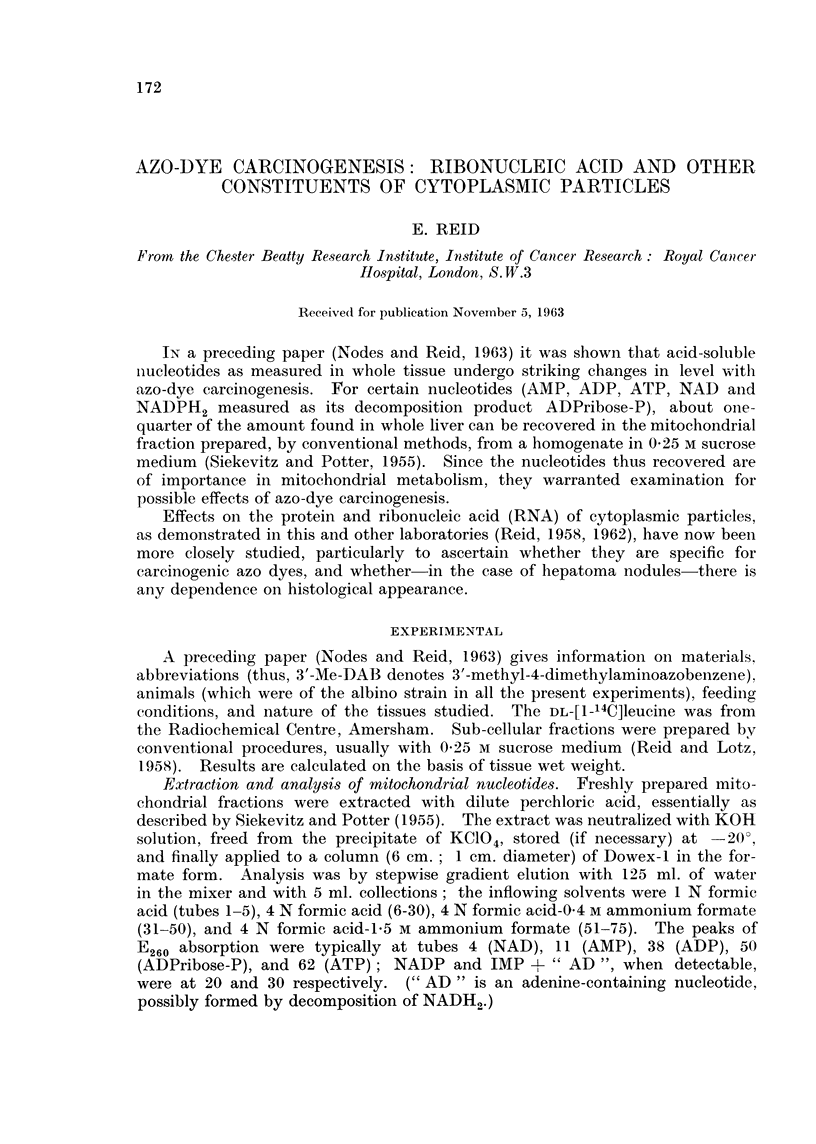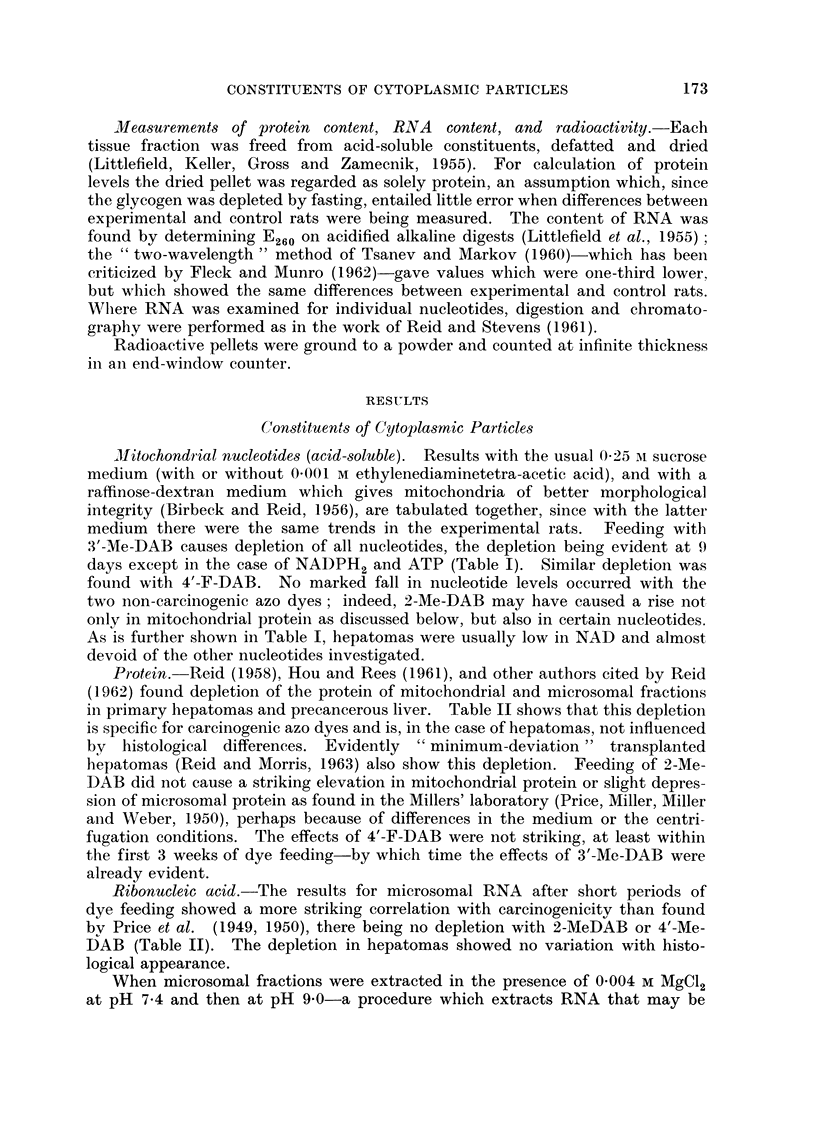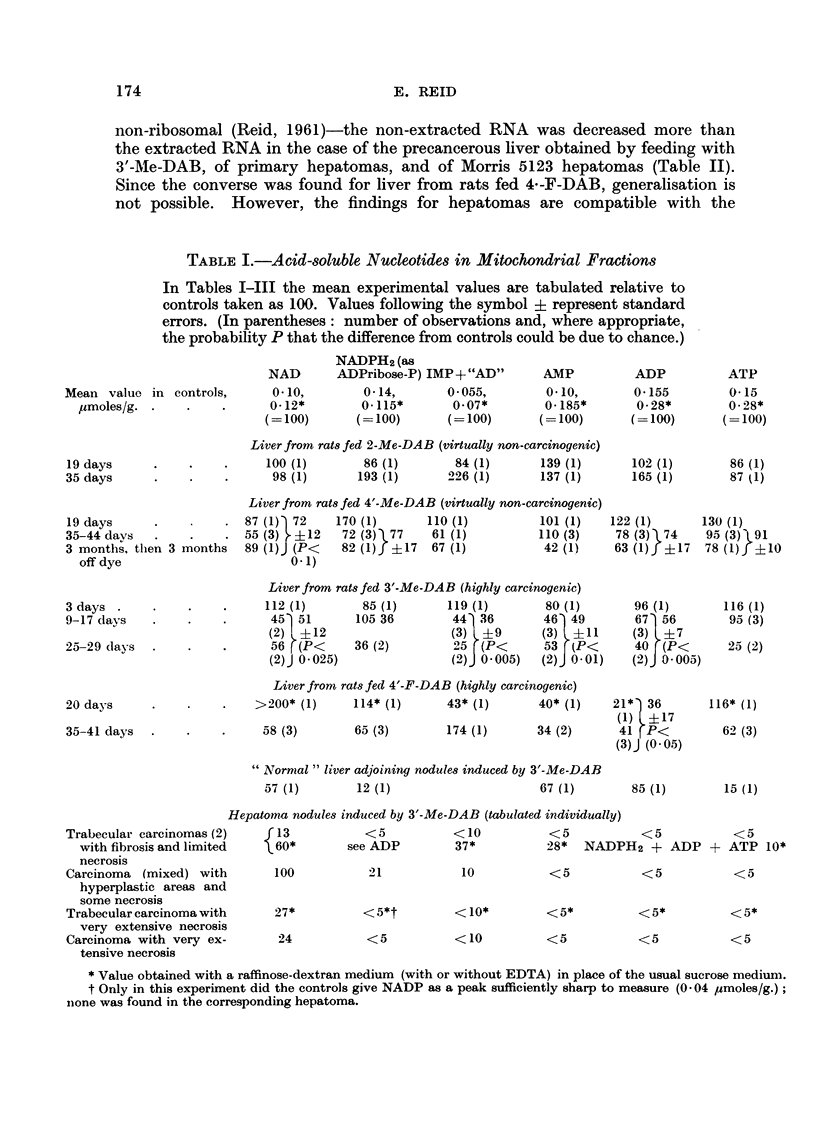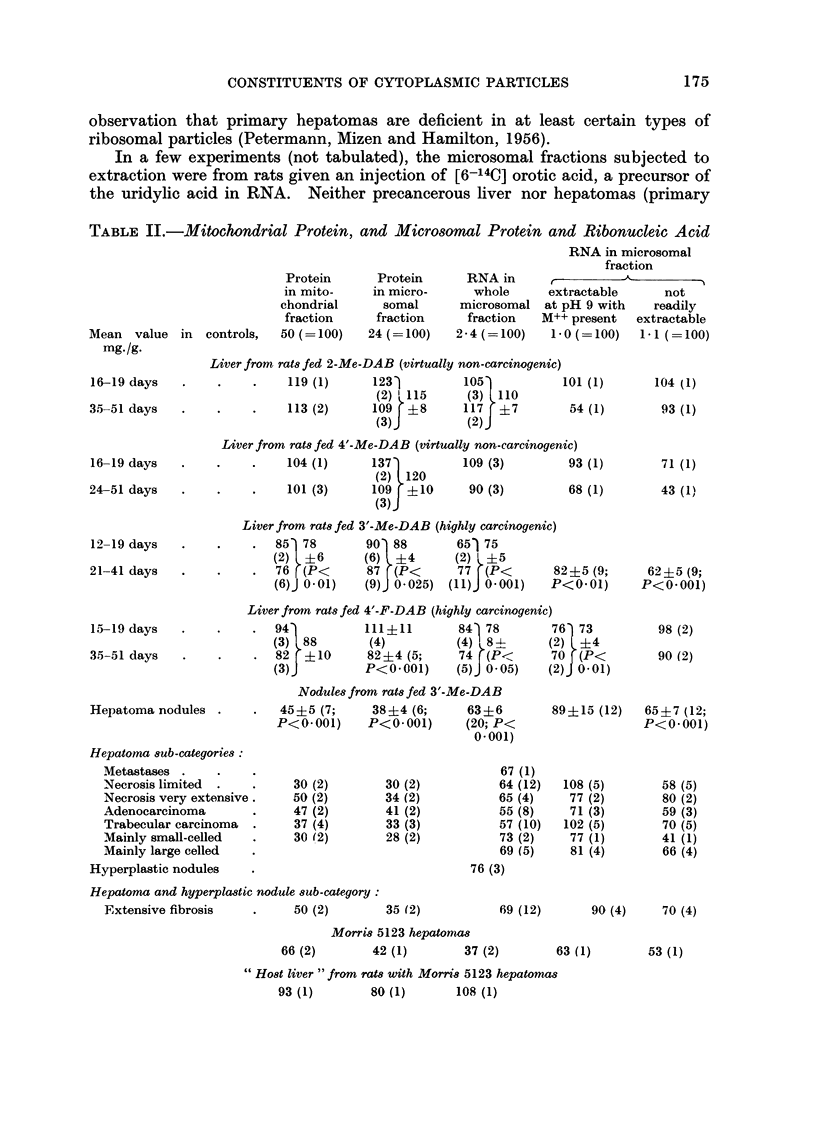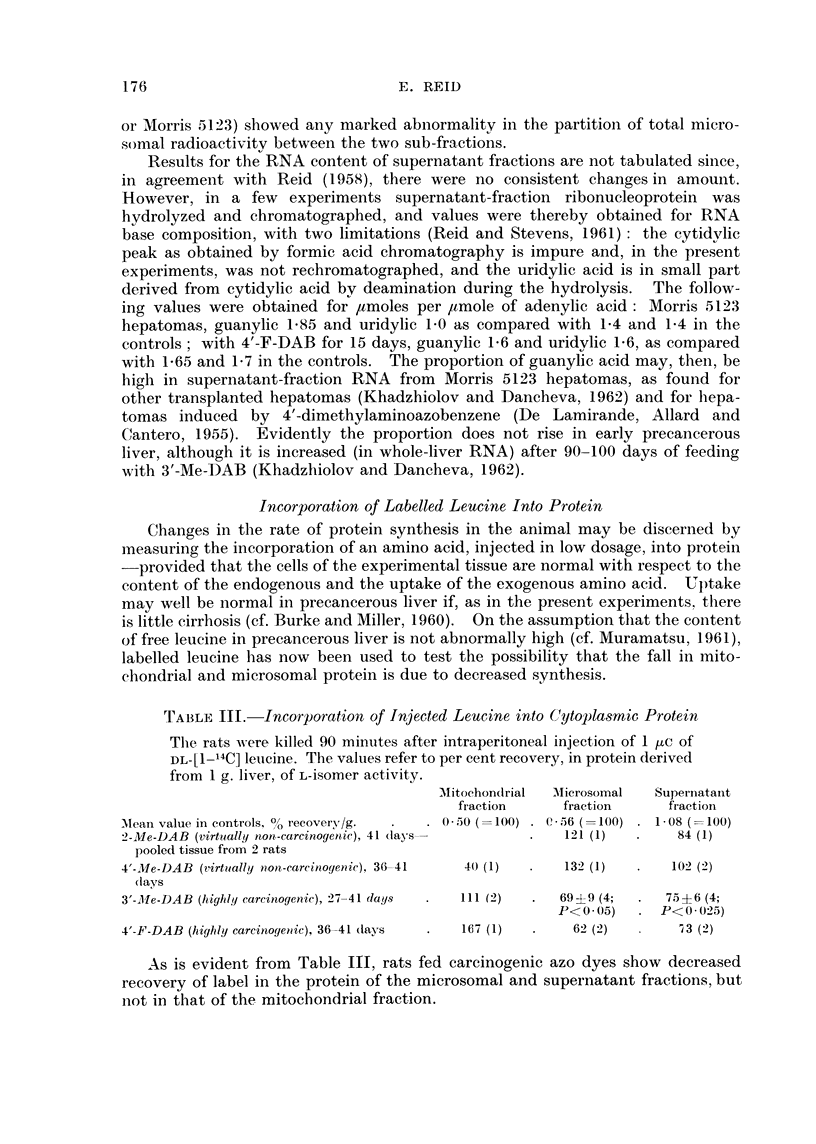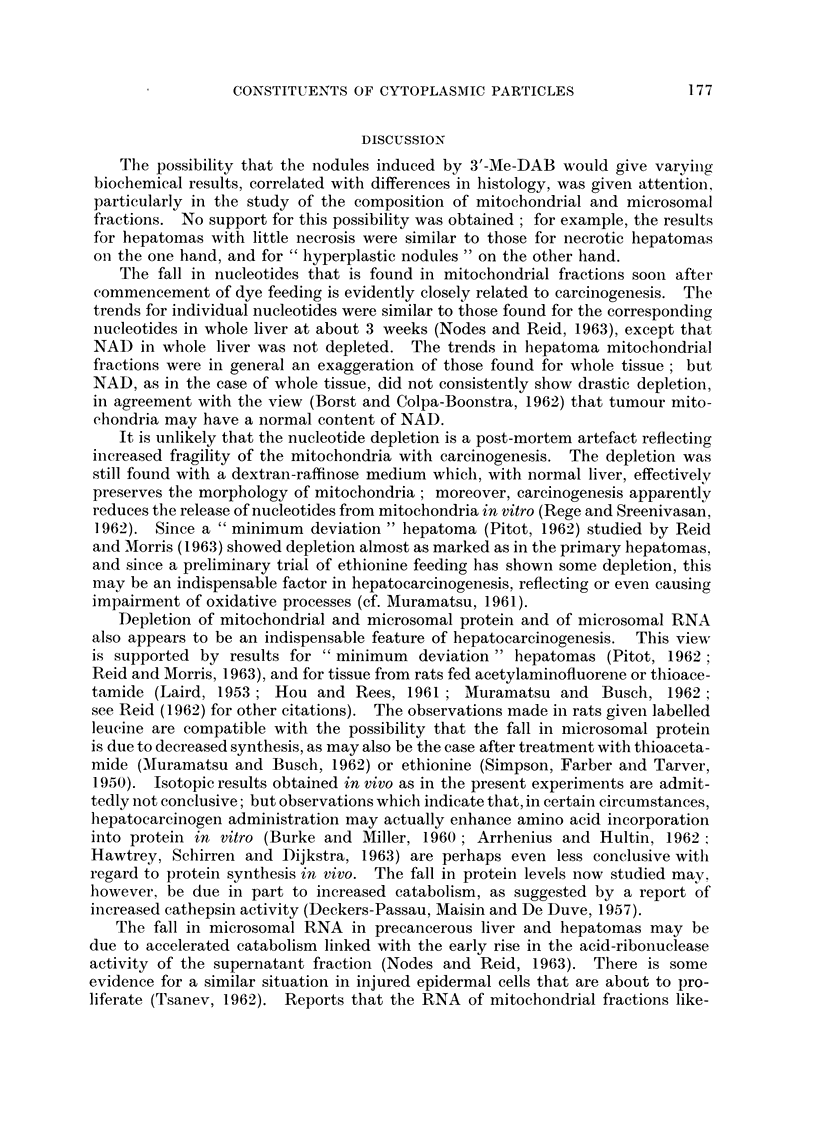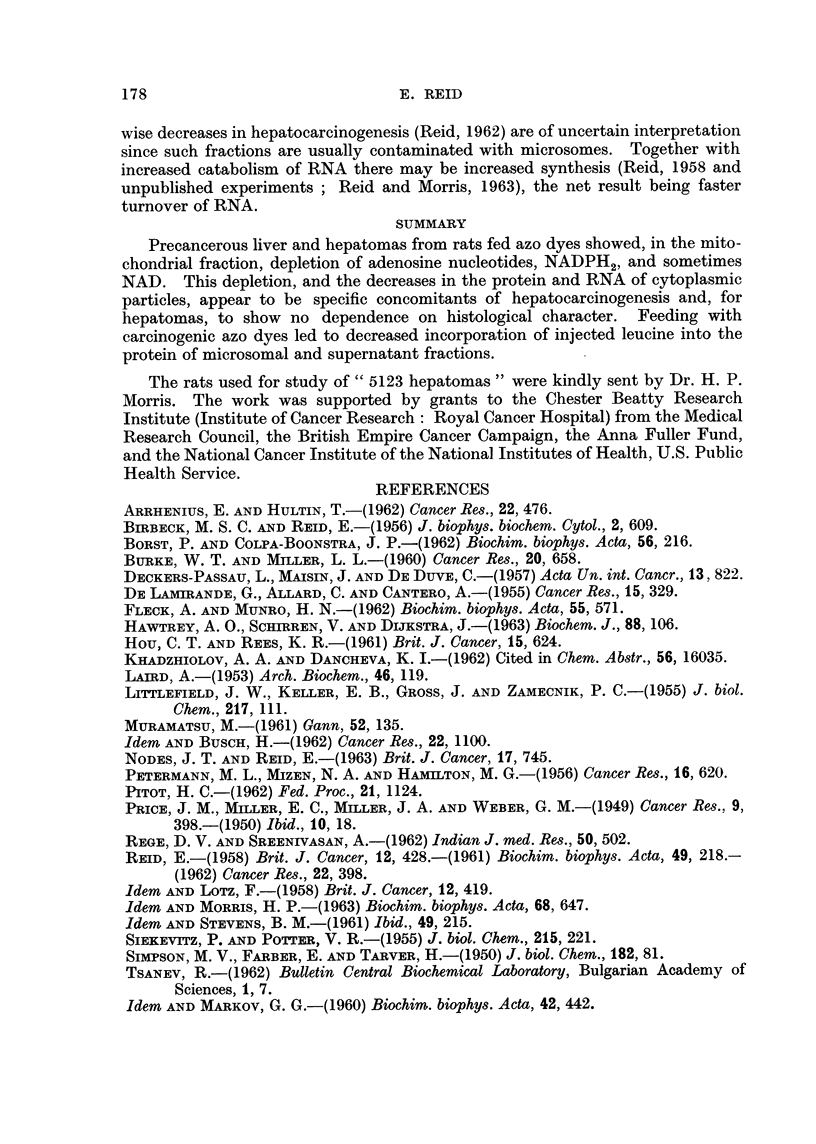# Azo-Dye Carcinogenesis: Ribonucleic Acid and Other Constituents of Cytoplasmic Particles

**DOI:** 10.1038/bjc.1964.20

**Published:** 1964-03

**Authors:** E. Reid


### AZO-DYE CARCINOGENESIS: RIBONUCLEIC ACID AND OTHER CONSTITUENTS OF CYTOPLASMIC PARTICLES

E. REID

*From the Chester Beatty Research Institute, Institute of Cancer Research: Royal Cancer Hospital, London, S.W.3*

Received for publication November 5, 1963

In a preceding paper (Nodes and Reid, 1963) it was shown that acid-soluble nucleotides as measured in whole tissue undergo striking changes in level with azo-dye carcinogenesis. For certain nucleotides (AMP, ADP, ATP, NAD and $NADPH_2$ measured as its decomposition product ADPribose-P), about one-quarter of the amount found in whole liver can be recovered in the mitochondrial fraction prepared, by conventional methods, from a homogenate in 0·25 M sucrose medium (Siekevitz and Potter, 1955). Since the nucleotides thus recovered are of importance in mitochondrial metabolism, they warranted examination for possible effects of azo-dye carcinogenesis.

Effects on the protein and ribonucleic acid (RNA) of cytoplasmic particles, as demonstrated in this and other laboratories (Reid, 1958, 1962), have now been more closely studied, particularly to ascertain whether they are specific for carcinogenic azo dyes, and whether—in the case of hepatoma nodules—there is any dependence on histological appearance.

### EXPERIMENTAL

A preceding paper (Nodes and Reid, 1963) gives information on materials, abbreviations (thus, 3′-Me-DAB denotes 3′-methyl-4-dimethylaminoazobenzene), animals (which were of the albino strain in all the present experiments), feeding conditions, and nature of the tissues studied. The DL-[1-$^{14}$C]leucine was from the Radiochemical Centre, Amersham. Sub-cellular fractions were prepared by conventional procedures, usually with 0·25 M sucrose medium (Reid and Lotz, 1958). Results are calculated on the basis of tissue wet weight.

*Extraction and analysis of mitochondrial nucleotides.* Freshly prepared mitochondrial fractions were extracted with dilute perchloric acid, essentially as described by Siekevitz and Potter (1955). The extract was neutralized with KOH solution, freed from the precipitate of $KClO_4$, stored (if necessary) at $-20°$, and finally applied to a column (6 cm.; 1 cm. diameter) of Dowex-1 in the formate form. Analysis was by stepwise gradient elution with 125 ml. of water in the mixer and with 5 ml. collections; the inflowing solvents were 1 N formic acid (tubes 1–5), 4 N formic acid (6-30), 4 N formic acid-0·4 M ammonium formate (31–50), and 4 N formic acid-1·5 M ammonium formate (51–75). The peaks of $E_{260}$ absorption were typically at tubes 4 (NAD), 11 (AMP), 38 (ADP), 50 (ADPribose-P), and 62 (ATP); NADP and IMP + " AD ", when detectable, were at 20 and 30 respectively. (" AD " is an adenine-containing nucleotide, possibly formed by decomposition of $NADH_2$.)

*Measurements of protein content, RNA content, and radioactivity.*—Each tissue fraction was freed from acid-soluble constituents, defatted and dried (Littlefield, Keller, Gross and Zamecnik, 1955). For calculation of protein levels the dried pellet was regarded as solely protein, an assumption which, since the glycogen was depleted by fasting, entailed little error when differences between experimental and control rats were being measured. The content of RNA was found by determining $E_{260}$ on acidified alkaline digests (Littlefield *et al.*, 1955); the " two-wavelength " method of Tsanev and Markov (1960)—which has been criticized by Fleck and Munro (1962)—gave values which were one-third lower, but which showed the same differences between experimental and control rats. Where RNA was examined for individual nucleotides, digestion and chromatography were performed as in the work of Reid and Stevens (1961).

Radioactive pellets were ground to a powder and counted at infinite thickness in an end-window counter.

### RESULTS

### *Constituents of Cytoplasmic Particles*

*Mitochondrial nucleotides (acid-soluble).* Results with the usual 0·25 M sucrose medium (with or without 0·001 M ethylenediaminetetra-acetic acid), and with a raffinose-dextran medium which gives mitochondria of better morphological integrity (Birbeck and Reid, 1956), are tabulated together, since with the latter medium there were the same trends in the experimental rats. Feeding with 3′-Me-DAB causes depletion of all nucleotides, the depletion being evident at 9 days except in the case of $NADPH_2$ and ATP (Table I). Similar depletion was found with 4′-F-DAB. No marked fall in nucleotide levels occurred with the two non-carcinogenic azo dyes; indeed, 2-Me-DAB may have caused a rise not only in mitochondrial protein as discussed below, but also in certain nucleotides. As is further shown in Table I, hepatomas were usually low in NAD and almost devoid of the other nucleotides investigated.

*Protein.*—Reid (1958), Hou and Rees (1961), and other authors cited by Reid (1962) found depletion of the protein of mitochondrial and microsomal fractions in primary hepatomas and precancerous liver. Table II shows that this depletion is specific for carcinogenic azo dyes and is, in the case of hepatomas, not influenced by histological differences. Evidently " minimum-deviation " transplanted hepatomas (Reid and Morris, 1963) also show this depletion. Feeding of 2-Me-DAB did not cause a striking elevation in mitochondrial protein or slight depression of microsomal protein as found in the Millers' laboratory (Price, Miller, Miller and Weber, 1950), perhaps because of differences in the medium or the centrifugation conditions. The effects of 4′-F-DAB were not striking, at least within the first 3 weeks of dye feeding—by which time the effects of 3′-Me-DAB were already evident.

*Ribonucleic acid.*—The results for microsomal RNA after short periods of dye feeding showed a more striking correlation with carcinogenicity than found by Price *et al.* (1949, 1950), there being no depletion with 2-MeDAB or 4′-Me-DAB (Table II). The depletion in hepatomas showed no variation with histological appearance.

When microsomal fractions were extracted in the presence of 0·004 M $MgCl_2$ at pH 7·4 and then at pH 9·0—a procedure which extracts RNA that may be non-ribosomal (Reid, 1961)—the non-extracted RNA was decreased more than the extracted RNA in the case of the precancerous liver obtained by feeding with 3′-Me-DAB, of primary hepatomas, and of Morris 5123 hepatomas (Table II). Since the converse was found for liver from rats fed 4·-F-DAB, generalisation is not possible. However, the findings for hepatomas are compatible with the

TABLE I.—*Acid-soluble Nucleotides in Mitochondrial Fractions*

In Tables I–III the mean experimental values are tabulated relative to controls taken as 100. Values following the symbol ± represent standard errors. (In parentheses : number of observations and, where appropriate, the probability *P* that the difference from controls could be due to chance.)

| | NAD | $NADPH_2$ (as ADPribose-P) | IMP+"AD" | AMP | ADP | ATP |
|---|---|---|---|---|---|---|
| Mean value in controls, μmoles/g. . . . | 0·10, 0·12* (=100) | 0·14, 0·115* (=100) | 0·055, 0·07* (=100) | 0·10, 0·185* (=100) | 0·155 0·28* (=100) | 0·15 0·28* (=100) |
| | *Liver from rats fed 2-Me-DAB (virtually non-carcinogenic)* | | | | | |
| 19 days . . . | 100 (1) | 86 (1) | 84 (1) | 139 (1) | 102 (1) | 86 (1) |
| 35 days . . . | 98 (1) | 193 (1) | 226 (1) | 137 (1) | 165 (1) | 87 (1) |
| | *Liver from rats fed 4′-Me-DAB (virtually non-carcinogenic)* | | | | | |
| 19 days . . . | 87 (1) } 72 | 170 (1) | 110 (1) | 101 (1) | 122 (1) | 130 (1) |
| 35–44 days . . . | 55 (3) } ±12 | 72 (3) } 77 | 61 (1) | 110 (3) | 78 (3) } 74 | 95 (3) } 91 |
| 3 months, then 3 months off dye | 89 (1) } (*P*< 0·1) | 82 (1) } ±17 | 67 (1) | 42 (1) | 63 (1) } ±17 | 78 (1) } ±10 |
| | *Liver from rats fed 3′-Me-DAB (highly carcinogenic)* | | | | | |
| 3 days . . . . | 112 (1) | 85 (1) | 119 (1) | 80 (1) | 96 (1) | 116 (1) |
| 9–17 days . . . | 45 (2) } 51 ±12 | 105 36 | 44 (3) } 36 ±9 | 46 (3) } 49 ±11 | 67 (3) } 56 ±7 | 95 (3) |
| 25–29 days . . . | 56 (2) } (*P*< 0·025) | 36 (2) | 25 (2) } (*P*< 0·005) | 53 (2) } (*P*< 0·01) | 40 (2) } (*P*< 0·005) | 25 (2) |
| | *Liver from rats fed 4′-F-DAB (highly carcinogenic)* | | | | | |
| 20 days . . . | >200* (1) | 114* (1) | 43* (1) | 40* (1) | 21* (1) } 36 ±17 | 116* (1) |
| 35–41 days . . . | 58 (3) | 65 (3) | 174 (1) | 34 (2) | 41 (3) } *P*< (0·05) | 62 (3) |
| | *" Normal " liver adjoining nodules induced by 3′-Me-DAB* | | | | | |
| | 57 (1) | 12 (1) | | 67 (1) | 85 (1) | 15 (1) |
| | *Hepatoma nodules induced by 3′-Me-DAB (tabulated individually)* | | | | | |
| Trabecular carcinomas (2) with fibrosis and limited necrosis | {13 60* | <5 see ADP | <10 37* | <5 28* | <5 $NADPH_2$ + ADP + | <5 ATP 10* |
| Carcinoma (mixed) with hyperplastic areas and some necrosis | 100 | 21 | 10 | <5 | <5 | <5 |
| Trabecular carcinoma with very extensive necrosis | 27* | <5*† | <10* | <5* | <5* | <5* |
| Carcinoma with very extensive necrosis | 24 | <5 | <10 | <5 | <5 | <5 |

* Value obtained with a raffinose-dextran medium (with or without EDTA) in place of the usual sucrose medium.
† Only in this experiment did the controls give NADP as a peak sufficiently sharp to measure (0·04 μmoles/g.) ; none was found in the corresponding hepatoma.

observation that primary hepatomas are deficient in at least certain types of ribosomal particles (Petermann, Mizen and Hamilton, 1956).

In a few experiments (not tabulated), the microsomal fractions subjected to extraction were from rats given an injection of [$6-{}^{14}C$] orotic acid, a precursor of the uridylic acid in RNA. Neither precancerous liver nor hepatomas (primary

TABLE II.—*Mitochondrial Protein, and Microsomal Protein and Ribonucleic Acid*

| | Protein in mito-chondrial fraction | Protein in micro-somal fraction | RNA in whole microsomal fraction | RNA in microsomal fraction: extractable at pH 9 with $M^{++}$ present | RNA in microsomal fraction: not readily extractable |
|---|---|---|---|---|---|
| Mean value in controls, mg./g. | 50 (=100) | 24 (=100) | 2·4 (=100) | 1·0 (=100) | 1·1 (=100) |
| *Liver from rats fed 2-Me-DAB (virtually non-carcinogenic)* | | | | | |
| 16–19 days | 119 (1) | 123 (2) } 115 ±8 | 105 (3) } 110 ±7 | 101 (1) | 104 (1) |
| 35–51 days | 113 (2) | 109 (3) | 117 (2) | 54 (1) | 93 (1) |
| *Liver from rats fed 4′-Me-DAB (virtually non-carcinogenic)* | | | | | |
| 16–19 days | 104 (1) | 137 (2) } 120 ±10 | 109 (3) | 93 (1) | 71 (1) |
| 24–51 days | 101 (3) | 109 (3) | 90 (3) | 68 (1) | 43 (1) |
| *Liver from rats fed 3′-Me-DAB (highly carcinogenic)* | | | | | |
| 12–19 days | 85 (2) } 78 ±6 (P< 0·01) | 90 (6) } 88 ±4 (P< 0·025) | 65 (2) } 75 ±5 (P< 0·001) | | |
| 21–41 days | 76 (6) | 87 (9) | 77 (11) | 82±5 (9; P<0·01) | 62±5 (9; P<0·001) |
| *Liver from rats fed 4′-F-DAB (highly carcinogenic)* | | | | | |
| 15–19 days | 94 (3) } 88 ±10 | 111±11 (4) | 84 (4) } 78 8± (P< 0·05) | 76 (2) } 73 ±4 (P< 0·01) | 98 (2) |
| 35–51 days | 82 (3) | 82±4 (5; P<0·001) | 74 (5) | 70 (2) | 90 (2) |
| *Nodules from rats fed 3′-Me-DAB* | | | | | |
| Hepatoma nodules | 45±5 (7; P<0·001) | 38±4 (6; P<0·001) | 63±6 (20; P< 0·001) | 89±15 (12) | 65±7 (12; P<0·001) |
| *Hepatoma sub-categories :* | | | | | |
| Metastases | | | 67 (1) | | |
| Necrosis limited | 30 (2) | 30 (2) | 64 (12) | 108 (5) | 58 (5) |
| Necrosis very extensive | 50 (2) | 34 (2) | 65 (4) | 77 (2) | 80 (2) |
| Adenocarcinoma | 47 (2) | 41 (2) | 55 (8) | 71 (3) | 59 (3) |
| Trabecular carcinoma | 37 (4) | 33 (3) | 57 (10) | 102 (5) | 70 (5) |
| Mainly small-celled | 30 (2) | 28 (2) | 73 (2) | 77 (1) | 41 (1) |
| Mainly large celled | | | 69 (5) | 81 (4) | 66 (4) |
| Hyperplastic nodules | | | 76 (3) | | |
| *Hepatoma and hyperplastic nodule sub-category :* | | | | | |
| Extensive fibrosis | 50 (2) | 35 (2) | 69 (12) | 90 (4) | 70 (4) |
| *Morris 5123 hepatomas* | | | | | |
| | 66 (2) | 42 (1) | 37 (2) | 63 (1) | 53 (1) |
| *" Host liver " from rats with Morris 5123 hepatomas* | | | | | |
| | 93 (1) | 80 (1) | 108 (1) | | |

or Morris 5123) showed any marked abnormality in the partition of total microsomal radioactivity between the two sub-fractions.

Results for the RNA content of supernatant fractions are not tabulated since, in agreement with Reid (1958), there were no consistent changes in amount. However, in a few experiments supernatant-fraction ribonucleoprotein was hydrolyzed and chromatographed, and values were thereby obtained for RNA base composition, with two limitations (Reid and Stevens, 1961) : the cytidylic peak as obtained by formic acid chromatography is impure and, in the present experiments, was not rechromatographed, and the uridylic acid is in small part derived from cytidylic acid by deamination during the hydrolysis. The following values were obtained for $\mu$moles per $\mu$mole of adenylic acid : Morris 5123 hepatomas, guanylic 1·85 and uridylic 1·0 as compared with 1·4 and 1·4 in the controls ; with 4′-F-DAB for 15 days, guanylic 1·6 and uridylic 1·6, as compared with 1·65 and 1·7 in the controls. The proportion of guanylic acid may, then, be high in supernatant-fraction RNA from Morris 5123 hepatomas, as found for other transplanted hepatomas (Khadzhiolov and Dancheva, 1962) and for hepatomas induced by 4′-dimethylaminoazobenzene (De Lamirande, Allard and Cantero, 1955). Evidently the proportion does not rise in early precancerous liver, although it is increased (in whole-liver RNA) after 90–100 days of feeding with 3′-Me-DAB (Khadzhiolov and Dancheva, 1962).

### *Incorporation of Labelled Leucine Into Protein*

Changes in the rate of protein synthesis in the animal may be discerned by measuring the incorporation of an amino acid, injected in low dosage, into protein —provided that the cells of the experimental tissue are normal with respect to the content of the endogenous and the uptake of the exogenous amino acid. Uptake may well be normal in precancerous liver if, as in the present experiments, there is little cirrhosis (cf. Burke and Miller, 1960). On the assumption that the content of free leucine in precancerous liver is not abnormally high (cf. Muramatsu, 1961), labelled leucine has now been used to test the possibility that the fall in mitochondrial and microsomal protein is due to decreased synthesis.

TABLE III.—*Incorporation of Injected Leucine into Cytoplasmic Protein*

The rats were killed 90 minutes after intraperitoneal injection of 1 $\mu$c of DL-[1–$^{14}$C] leucine. The values refer to per cent recovery, in protein derived from 1 g. liver, of L-isomer activity.

| | Mitochondrial fraction | Microsomal fraction | Supernatant fraction |
|---|---|---|---|
| Mean value in controls, % recovery/g. | 0·50 (=100) | 0·56 (=100) | 1·08 (=100) |
| 2-*Me-DAB* (*virtually non-carcinogenic*), 41 days—pooled tissue from 2 rats | | 121 (1) | 84 (1) |
| 4′-*Me-DAB* (*virtually non-carcinogenic*), 36–41 days | 40 (1) | 132 (1) | 102 (2) |
| 3′-*Me-DAB* (*highly carcinogenic*), 27–41 *days* | 111 (2) | 69±9 (4; $P<0·05$) | 75±6 (4; $P<0·025$) |
| 4′-*F-DAB* (*highly carcinogenic*), 36–41 days | 167 (1) | 62 (2) | 73 (2) |

As is evident from Table III, rats fed carcinogenic azo dyes show decreased recovery of label in the protein of the microsomal and supernatant fractions, but not in that of the mitochondrial fraction.

### DISCUSSION

The possibility that the nodules induced by 3′-Me-DAB would give varying biochemical results, correlated with differences in histology, was given attention, particularly in the study of the composition of mitochondrial and microsomal fractions. No support for this possibility was obtained ; for example, the results for hepatomas with little necrosis were similar to those for necrotic hepatomas on the one hand, and for " hyperplastic nodules " on the other hand.

The fall in nucleotides that is found in mitochondrial fractions soon after commencement of dye feeding is evidently closely related to carcinogenesis. The trends for individual nucleotides were similar to those found for the corresponding nucleotides in whole liver at about 3 weeks (Nodes and Reid, 1963), except that NAD in whole liver was not depleted. The trends in hepatoma mitochondrial fractions were in general an exaggeration of those found for whole tissue ; but NAD, as in the case of whole tissue, did not consistently show drastic depletion, in agreement with the view (Borst and Colpa-Boonstra, 1962) that tumour mitochondria may have a normal content of NAD.

It is unlikely that the nucleotide depletion is a post-mortem artefact reflecting increased fragility of the mitochondria with carcinogenesis. The depletion was still found with a dextran-raffinose medium which, with normal liver, effectively preserves the morphology of mitochondria ; moreover, carcinogenesis apparently reduces the release of nucleotides from mitochondria *in vitro* (Rege and Sreenivasan, 1962). Since a " minimum deviation " hepatoma (Pitot, 1962) studied by Reid and Morris (1963) showed depletion almost as marked as in the primary hepatomas, and since a preliminary trial of ethionine feeding has shown some depletion, this may be an indispensable factor in hepatocarcinogenesis, reflecting or even causing impairment of oxidative processes (cf. Muramatsu, 1961).

Depletion of mitochondrial and microsomal protein and of microsomal RNA also appears to be an indispensable feature of hepatocarcinogenesis. This view is supported by results for " minimum deviation " hepatomas (Pitot, 1962 ; Reid and Morris, 1963), and for tissue from rats fed acetylaminofluorene or thioacetamide (Laird, 1953 ; Hou and Rees, 1961 ; Muramatsu and Busch, 1962 ; see Reid (1962) for other citations). The observations made in rats given labelled leucine are compatible with the possibility that the fall in microsomal protein is due to decreased synthesis, as may also be the case after treatment with thioacetamide (Muramatsu and Busch, 1962) or ethionine (Simpson, Farber and Tarver, 1950). Isotopic results obtained *in vivo* as in the present experiments are admittedly not conclusive; but observations which indicate that, in certain circumstances, hepatocarcinogen administration may actually enhance amino acid incorporation into protein *in vitro* (Burke and Miller, 1960 ; Arrhenius and Hultin, 1962 ; Hawtrey, Schirren and Dijkstra, 1963) are perhaps even less conclusive with regard to protein synthesis *in vivo*. The fall in protein levels now studied may, however, be due in part to increased catabolism, as suggested by a report of increased cathepsin activity (Deckers-Passau, Maisin and De Duve, 1957).

The fall in microsomal RNA in precancerous liver and hepatomas may be due to accelerated catabolism linked with the early rise in the acid-ribonuclease activity of the supernatant fraction (Nodes and Reid, 1963). There is some evidence for a similar situation in injured epidermal cells that are about to proliferate (Tsanev, 1962). Reports that the RNA of mitochondrial fractions likewise decreases in hepatocarcinogenesis (Reid, 1962) are of uncertain interpretation since such fractions are usually contaminated with microsomes. Together with increased catabolism of RNA there may be increased synthesis (Reid, 1958 and unpublished experiments ; Reid and Morris, 1963), the net result being faster turnover of RNA.

SUMMARY

Precancerous liver and hepatomas from rats fed azo dyes showed, in the mitochondrial fraction, depletion of adenosine nucleotides, $NADPH_2$, and sometimes NAD. This depletion, and the decreases in the protein and RNA of cytoplasmic particles, appear to be specific concomitants of hepatocarcinogenesis and, for hepatomas, to show no dependence on histological character. Feeding with carcinogenic azo dyes led to decreased incorporation of injected leucine into the protein of microsomal and supernatant fractions.

The rats used for study of " 5123 hepatomas " were kindly sent by Dr. H. P. Morris. The work was supported by grants to the Chester Beatty Research Institute (Institute of Cancer Research : Royal Cancer Hospital) from the Medical Research Council, the British Empire Cancer Campaign, the Anna Fuller Fund, and the National Cancer Institute of the National Institutes of Health, U.S. Public Health Service.

REFERENCES

Arrhenius, E. and Hultin, T.—(1962) *Cancer Res.*, **22**, 476.
Birbeck, M. S. C. and Reid, E.—(1956) *J. biophys. biochem. Cytol.*, **2**, 609.
Borst, P. and Colpa-Boonstra, J. P.—(1962) *Biochim. biophys. Acta*, **56**, 216.
Burke, W. T. and Miller, L. L.—(1960) *Cancer Res.*, **20**, 658.
Deckers-Passau, L., Maisin, J. and De Duve, C.—(1957) *Acta Un. int. Cancr.*, **13**, 822.
De Lamirande, G., Allard, C. and Cantero, A.—(1955) *Cancer Res.*, **15**, 329.
Fleck, A. and Munro, H. N.—(1962) *Biochim. biophys. Acta*, **55**, 571.
Hawtrey, A. O., Schirren, V. and Dijkstra, J.—(1963) *Biochem. J.*, **88**, 106.
Hou, C. T. and Rees, K. R.—(1961) *Brit. J. Cancer*, **15**, 624.
Khadzhiolov, A. A. and Dancheva, K. I.—(1962) Cited in *Chem. Abstr.*, **56**, 16035.
Laird, A.—(1953) *Arch. Biochem.*, **46**, 119.
Littlefield, J. W., Keller, E. B., Gross, J. and Zamecnik, P. C.—(1955) *J. biol. Chem.*, **217**, 111.
Muramatsu, M.—(1961) *Gann*, **52**, 135.
*Idem* and Busch, H.—(1962) *Cancer Res.*, **22**, 1100.
Nodes, J. T. and Reid, E.—(1963) *Brit. J. Cancer*, **17**, 745.
Petermann, M. L., Mizen, N. A. and Hamilton, M. G.—(1956) *Cancer Res.*, **16**, 620.
Pitot, H. C.—(1962) *Fed. Proc.*, **21**, 1124.
Price, J. M., Miller, E. C., Miller, J. A. and Weber, G. M.—(1949) *Cancer Res.*, **9**, 398.—(1950) *Ibid.*, **10**, 18.
Rege, D. V. and Sreenivasan, A.—(1962) *Indian J. med. Res.*, **50**, 502.
Reid, E.—(1958) *Brit. J. Cancer*, **12**, 428.—(1961) *Biochim. biophys. Acta*, **49**, 218.—(1962) *Cancer Res.*, **22**, 398.
*Idem* and Lotz, F.—(1958) *Brit. J. Cancer*, **12**, 419.
*Idem* and Morris, H. P.—(1963) *Biochim. biophys. Acta*, **68**, 647.
*Idem* and Stevens, B. M.—(1961) *Ibid.*, **49**, 215.
Siekevitz, P. and Potter, V. R.—(1955) *J. biol. Chem.*, **215**, 221.
Simpson, M. V., Farber, E. and Tarver, H.—(1950) *J. biol. Chem.*, **182**, 81.
Tsanev, R.—(1962) *Bulletin Central Biochemical Laboratory*, Bulgarian Academy of Sciences, **1**, 7.
*Idem* and Markov, G. G.—(1960) *Biochim. biophys. Acta*, **42**, 442.